# Molecular Epidemiology of Global Carbapenemase-Producing *Citrobacter* spp. (2015–2017)

**DOI:** 10.1128/spectrum.04144-22

**Published:** 2023-02-27

**Authors:** Diego Nobrega, Gisele Peirano, Yasufumi Matsumura, Johann D. D. Pitout

**Affiliations:** a Faculty of Veterinary Medicine, University of Calgary, Calgary, Alberta, Canada; b Alberta Precision Laboratories, Calgary, Alberta, Canada; c Cummings School of Medicine, University of Calgary, Calgary, Alberta, Canada; d Kyoto University Graduate School of Medicine, Kyoto, Japan; e University of Pretoria, Pretoria, South Africa; Emory University School of Medicine

**Keywords:** carbapenemase-producing Enterobacterales, *Citrobacter* spp., molecular epidemiology, population-based surveillance, carbapenemase

## Abstract

The emergence of carbapenem resistance is a significant public health concern. The rate of infections caused by carbapenemase-producing *Citrobacter* spp., particularly C. freundii, is increasing. Concomitantly, comprehensive global genomic data on carbapenemase-producing *Citrobacter* spp. are scarce. We used short read whole-genome sequencing to describe the molecular epidemiology and international distribution of eighty-six carbapenemase-producing *Citrobacter* spp. obtained from two surveillance programs (2015 to 17). The common carbapenemases were KPC-2 (26%), VIM-1 (17%), IMP-4 (14%) and NDM-1 (10%). C. freundii and C. portucalensis were the principal species. C. freundii consisted of multiple clones obtained mainly from Colombia (with KPC-2), the United States (with KPC-2, -3), and Italy (with VIM-1). Two dominant C. freundii clones were identified: ST98 was linked with *bla*_IMP-8_ from Taiwan and *bla*_KPC-2_ from the United States, and ST22 was linked with *bla*_KPC-2_ from Colombia and *bla*_VIM-1_ from Italy. C. portucalensis consisted mainly of two clones: ST493 with *bla*_IMP-4_ which was limited to Australia, and ST545 with *bla*_VIM-31_ which was limited to Turkey. Class I integron (In916) with *bla*_VIM-1_ was circulating between multiple sequence types (STs) in Italy, Poland, and Portugal. In73 with *bla*_IMP-8_ was circulating between various STs in Taiwan, while In809 with *bla*_IMP-4_ was circulating between different STs in Australia. The global carbapenemase-producing *Citrobacter* spp. population is dominated by diverse STs with different characteristics and varied geographical distribution and thus requires continued monitoring. Ongoing genomic surveillance should use methodologies able to distinguish between C. freundii and C. portucalensis.

**IMPORTANCE** *Citrobacter* spp. are gaining recognition as important causes of hospital-acquired infections in humans. Among *Citrobacter* spp., carbapenemase-producing strains are cause of utmost concern to health care services globally due to their ability to resist therapy with virtually any beta-lactam antibiotic. Here, we described the molecular characteristics of a global collection of carbapenemase-producing *Citrobacter* spp. C. freundii and *C. portucalensis* were the most common species among *Citrobacter* spp. with carbapenemases from this survey. Importantly, *C. portucalensis* was misidentified as C. freundii when using Vitek 2.0**/**MALDI-TOF MS (matrix-assisted laser desorption/ionization–time of flight mass spectrometry) phenotypic identification, which has important implications for future surveys. Among C. freundii, we identified two dominant clones: ST98 with *bla*_IMP-8_ from Taiwan and *bla*_KPC-2_ from the United States, and ST22 with *bla*_KPC-2_ from Colombia and *bla*_VIM-1_ from Italy. As for *C. portucalensis*, the dominant clones consisted of ST493 with *bla*_IMP-4_ from Australia and ST545 with *bla*_VIM-31_ from Turkey.

## INTRODUCTION

The continuous rise in infections caused by carbapenemase-producing Enterobacterales (CPE) is a cause of utmost concern to health care services globally (1). Infections caused by CPE are linked to high mortality rates in humans because these isolates are resistant to virtually all beta-lactam antibiotics and often harbor genetic determinants that confer resistance against other drug classes such as aminoglycosides and fluoroquinolones (2). CPEs often contain various plasmid-borne resistance genes, including KPCs, NDMs, and OXA-48-like carbapenemases (3).

Among the CPE, Klebsiella pneumoniae and Escherichia coli represent the main clinical load of infections (3). Nevertheless, *Citrobacter* spp. are gaining recognition as important causes of nosocomial infections (4). Recent evidence has suggested that the rate of infections caused by carbapenemase-producing *Citrobacter* spp., particularly C. freundii, is increasing (5, 6). In general, carbapenemase-producing *Citrobacter* spp. populations are genetically diverse (6), but a few sequence types (STs) such as ST22 and ST19 have recently emerged and demonstrated the potential to become dominant clones in health care settings (5).

To date, information regarding the molecular epidemiology of carbapenemase-producing *Citrobacter* spp., including its genetic diversity and genetic mechanisms of carbapenem resistance, is limited to regional or countrywide studies. From an epidemiological standpoint, such assessments are valuable to specific geographical areas, but fall short in providing a broader overview from a global perspective, which includes a description of global dominant STs and genetic mechanisms of carbapenem resistance. Here, we described the molecular characteristics of a global collection of carbapenemase-producing *Citrobacter* species isolates obtained systematically from two global surveillance programs. We report the geographical distribution of STs and provide an in-depth assessment of molecular mechanisms associated with carbapenem resistance in carbapenemase-producing *Citrobacter* spp.

## RESULTS

### Global distribution of carbapenemases.

Overall, high (≥20%), intermediate, or resistant (i.e., not susceptible [NS]) rates were found for ertapenem (100%), piperacillin-tazobactam (97%), meropenem (96%), ceftriaxone (93%), ceftazidime (92%), cefepime (92%), and trimethoprim-sulfamethoxazole (59%), followed by tetracycline (33%). Low NS rates (<10%) were found for ciprofloxacin (6%), gentamicin (8%), tobramycin (8%), and amikacin (2%). Isolates were susceptible to tigecycline and colistin. Gentamicin and tobramycin NS rates were associated with *aac(3′)-IIa* and amikacin rates with *armA*, *rmtC*, and *rmtD2*.

**FIG 1:**
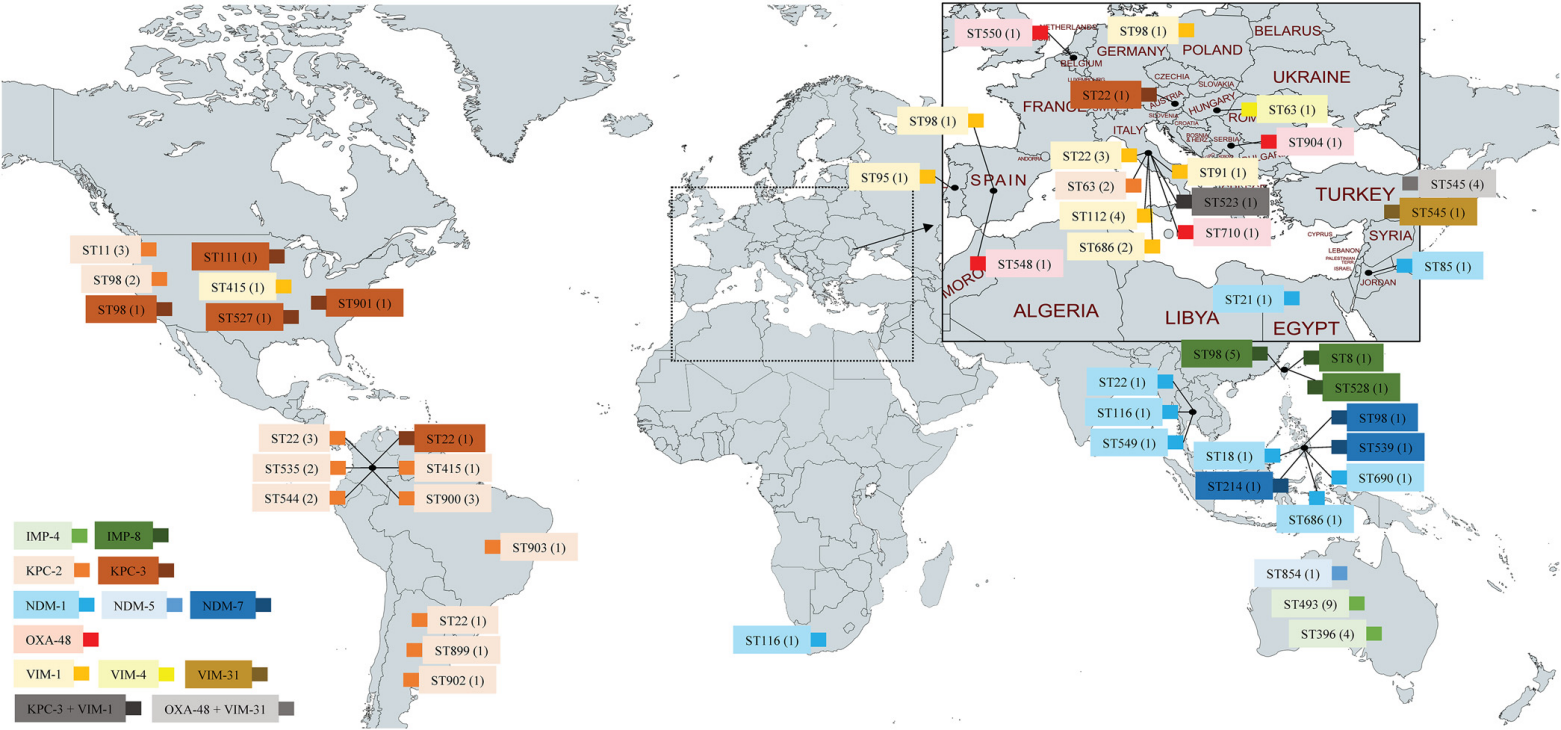
Global distribution of carbapenemase-positive *Citrobacter* spp., with an enlarged Mediterranean insert (map created with MapChart [https://www.mapchart.net/index.html]).

Overall, range of carbapenems MICs were similar for all STs. However, MICs were lower for OXA-48 isolates compared to other carbapenemases (OXA-48 [*n* = 4]: ertapenem = 2 to 32 μg/mL, imipenem = 2 to 16 μg/mL, and meropenem = 1 to 8 μg/mL; other carbapenemases [*n* = 82]: ertapenem = 16 to >64 μg/mL, imipenem = 8 to 32 μg/mL, and meropenem = 2 to 32 μg/mL). We detected 81 *Citrobacter* spp. which were positive for a single carbapenemase and 5 isolates harboring 2 different carbapenemases (Table S2). These isolates were obtained from the following countries: Argentina, Australia, Austria, Belgium, Brazil, Colombia, Egypt, Hungary, Italy, Jordan, Philippines, Poland, Portugal, Serbia, South Africa, Spain, Taiwan, Thailand, Turkey, and the United States. The most common carbapenemase groups included KPCs (*n* = 29), VIMs (*n* = 21), and IMPs (*n* = 20), followed by NDMs (*n* = 13) and OXA-48-like (*n* = 8). The OXA-48-like carbapenemases were identified as OXA-48. The most frequent individual carbapenemases consisted of KPC-2 (*n* = 22), VIM-1 (*n* = 15), IMP-4 (*n* = 13), and NDM-1 (*n* = 9) (Table S2). There was evidence of clustering at the continent level: KPCs were the most frequently detected carbapenemases in the Americas, whereas NDMs and IMPs were prevalent in Southeast Asia and Oceania, respectively (Fig. 1). VIMs were more frequent in Europe and OXA-48 in Turkey (Fig. 1).

### Species and sequence types.

**FIG 2:**
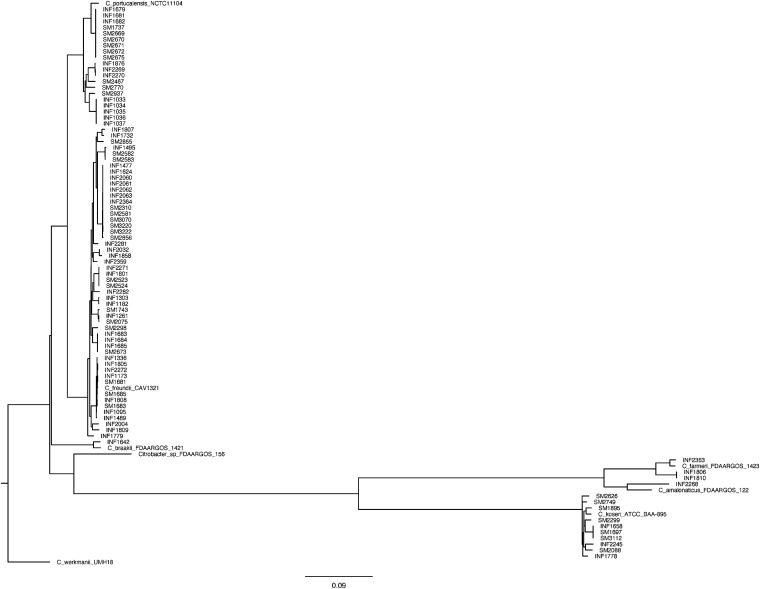
Phylogenetic tree of the different species, carbapenemases, countries, and sequence types among 86 *Citrobacter* spp. The tree was rooted on *C. werkmanii* UMH18. The kSNP tree was built based on 3,093 core single-nucleotide polymorphisms (SNPs) present in the 93 genomes. KPC, Klebsiella pneumoniae carbapenemase; NDM, New Delhi metallo-β-lactamase; OXA-48, oxacillinase; IMP, imipenemase; VIM, Verona imipenemase; ST, sequence type.

The following 6 species were identified (Table S2, Fig. 2): Citrobacter freundii (*n* = 51), Citrobacter portucalensis (*n* = 20), Citrobacter koseri (*n* = 10), Citrobacter farmeri (*n* = 3), Citrobacter amalonaticus, and Citrobacter braakii (*n* = 1 each). The *C. portucalensis* isolates were reported as C. freundii using MALDI-TOF MS (matrix-assisted laser desorption/ionization–time of flight mass spectrometry).

The most common carbapenemase among C. freundii (*n* = 51) was KPC-2 (27%), followed by VIM-1 (25%), IMP-8 (14%), KPC-3 (12%), NDM-1 (10%), IMP-4 (8%), NDM-7 (4%), and OXA-48 (2%). C. freundii consisted of 20 different STs which included two dominant clones, namely, ST22 (20%) and ST98 (22%) (Table 1, Fig. 2). The ST22 isolates (*n* = 10) showed a global distribution but clustered in Italy (*n* = 3 with VIM-1) and Colombia (*n* = 4 with KPC-2/3) (Fig. 1). The ST98 isolates (*n* = 11) also showed a global distribution but clustered in Taiwan (*n* = 5 with IMP-8) and the United States (*n* = 3 with KPC-2/3) (Fig. 1).

**TABLE 1:** Characteristics of dominant sequence types among carbapenemase-positive *Citrobacter* spp.

Characteristic	ST98 (*n* = 11)	ST22 (*n* = 10)	ST493 (*n* = 9)	ST545 (*n* = 5)	Other STs^*b*^ (*n* = 51)	All STs (*n* = 86)
Geographic location	Global^*c*^	Global^*d*^	Australia	Turkey	Global^*e*^	Global
*Citrobacter* species	*freundii*	*freundii*	*portucalensis*	*portucalensis*	Multiple^*f*^	Multiple
Carbapenemases, *n* (%)						
KPC-2	2 (18.2%)	4 (40%)	0 (0%)	0 (0%)	16 (31.4%)	22 (25.6%)
KPC-3	1 (9.1%)	2 (20%)	0 (0%)	0 (0%)	3 (5.9%)	6 (7%)
NDM-1	0 (0%)	1 (10%)	0 (0%)	0 (0%)	8 (15.7%)	9 (10.5%)
NDM-5	0 (0%)	0 (0%)	0 (0%)	0 (0%)	1 (2%)	1 (1.2%)
NDM-7	1 (9.1%)	0 (0%)	0 (0%)	0 (0%)	2 (3.9%)	3 (3.5%)
VIM-1	2 (18.2%)	3 (30%)	0 (0%)	0 (0%)	9 (17.6%)	14 (16.3%)
VIM-4	0 (0%)	0 (0%)	0 (0%)	0 (0%)	1 (2%)	1 (1.2%)
VIM-31	0 (0%)	0 (0%)	0 (0%)	1 (20%)	0 (0%)	1 (1.2%)
IMP-4	0 (0%)^*a*^	0 (0%)^*a*^	9 (100%)	0 (0%)^*a*^	4 (7.8%)	13 (15.1%)
IMP-8	5 (45.5%)	0 (0%)^*a*^	0 (0%)^*a*^	0 (0%)	2 (3.9%)	7 (8.1%)
OXA-48	0 (0%)	0 (0%)	0 (0%)	0 (0%)	4 (7.8%)	4 (4.7%)
KPC-3 + VIM-1	0 (0%)	0 (0%)	0 (0%)	0 (0%)	1 (2%)	1 (1.2%)
VIM-31 + OXA-48	0 (0%)^*a*^	0 (0%)^*a*^	0 (0%)^*a*^	4 (80%)	0 (0%)	4 (4.7%)
Other β-lactamases, *n* (%)						
CTX-M^*g*^	1 (9.1%)	2 (20%)	0 (0%)	0 (0%)	9 (17.6%)	12 (14%)
OXA^*h*^	4 (36.4%)^*a*^	2 (20%)^*a*^	8 (88.9%)	0 (0%)^*a*^	16 (31.4%)	30 (34.9%)
TEM-1	8 (72.7%)	2 (20%)^*a*^	9 (100%)	0 (0%)^*a*^	18 (35.3%)	37 (43%)
SHV-12	2 (18.2%)	3 (30%)	0 (0%)	0 (0%)	7 (13.7%)	12 (14%)

a Significantly different from the dominant sequence type(s) at the 5% level.

b Other STs (*n*) include the following: ST112 (4), ST396 (4), ST11 (3), ST63 (3), ST686 (3), ST900 (3), ST116 (2), ST415 (2), ST535 (2), ST544 (2), ST8 (1), ST18 (1), ST21 (1), ST85 (1), ST91 (1), ST95 (1), ST111 (1), ST214 (1), ST523 (1), ST527 (1), ST528 (1), ST539 (1), ST548 (1), ST549 (1), ST550 (1), ST690 (1), ST710 (1), ST854 (1), ST899 (1), ST901 (1), ST902 (1), ST903 (1), and ST904 (1).

c Taiwan (5), United States (3), Philippines (1), Poland (1), and Spain (1).

d Colombia (4), Italy (3), Argentina (1), Austria (1), and Thailand (1).

e Italy (11), Colombia (8), United States (7), Australia (5), Philippines (5), Argentina (2), Taiwan (2), Thailand (2), Belgium (1), Brazil (1), Egypt (1), Hungary (1), Jordan (1), Portugal (1), Serbia (1), South Africa (1), and Spain (1).

f C. freundii (30), C. koseri (10), C. portucalensis (6), C. farmeri (3), C. amalonaticus (1), and C. braakii (1).

g CTX-M-15 (6), CTX-M-12 (4), CTX-M-9 (2).

h OXA-1 (16), OXA-9 (8), OXA-10 (3), OXA-2 (2), OXA-1 + OXA-10 (1).

The most common carbapenemase among *C. portucalensis* (*n* = 20) was IMP-9 (45%), followed by VIM-31 (25%), OXA-48 (20%), NDM-1 (10%), and 5% for VIM-4 and NDM-7, respectively. *C. portucalensis* consisted of 6 different STs which included two dominant clones, namely, ST493 (45%) and ST545 (25%) (Table 1, Fig. 2). The ST493 isolates (*n* = 9) with IMP-4 were obtained from Australia and the ST545 isolates (*n* = 5) with VIM-31 were obtained from Turkey.

The most common carbapenemase among *C. koseri* (*n* = 10) was KPC-2 (*n* = 6) followed by KPC-3, NDM-1, NDM-5, and OXA-48 (1 isolate each). *C. koseri* consisted of 8 different STs with a global distribution (Table 1, Fig. 2). *C. farmeri* (*n* = 3) belonged to ST686 and was obtained from Italy (*n* = 2 with VIM-1) and the Philippines (*n* = 1 with NDM-1). *C. braakii* (*n* = 1) was obtained from Spain, belonged to ST548, and contained OXA-48. *C. amalonaticus* was obtained from Italy, belonged to ST710, and contained OXA-48.

### Dominant sequence types: antimicrobial resistance determinants and plasmid replicon types.

C. freundii ST98 were positive for IMP-8 (46%, 5 isolates), KPC-2 (18%, 2 isolates), VIM-1 (18%, 2 isolates), KPC-3 (9%, 1 isolate), and NDM-7 (9%, 1 isolate). The frequency of IMP-8 was higher in ST98 compared to other dominant STs (Table 1). IMP-8 positive ST98 were obtained from Taiwan and contained IncFII replicon types, which were absent among IMP-8-negative ST98. ST98 were positive for other antimicrobial resistance (AMR) determinants, including *aac(6′)-Ib-cr*, *aadA1*, *dfrA14*, *sul1*, and others (Table S3).

C. freundii ST22 were positive for KPC-2 (40%, 4 isolates), VIM-1 (30%, 3 isolates), KPC-3 (20%, 2 isolates), and NDM-1 (10%, 1 isolate) (Table 1). The three VIM-1 positive ST22 isolates were obtained from Italy and were positive for *aacA4*, *aphA15*, *aadA1b*, *catB2*, *dfrA1*, *sat2*, and *aadA1*. Two of the three VIM-1-positive isolates contained IncC replicons that were absent in VIM-1 negative ST22. No replicons were detected in the remaining isolate. ST22 with KPCs were obtained from Colombia (*n* = 4), Argentina, and Austria (*n* = 1 each) (Table 1). Different replicons were detected in KPC-positive ST22, including IncFII, IncX3, IncX5, and IncN (2 isolates each). Most of the KPC-positive ST22 also contained other AMR determinants, including *mphA* (5 out of 6 isolates), *tetD*, *arr3*, and *sul2* (4 isolates each). In addition, most ST22 (80%) were positive for *aac(6′)-Ib-cr*. The NDM-1-positive ST22 was obtained from Thailand and contained *qnrB4*, *tetA*, *sul1*, *sul2*, *aac(6′)-Ib-cr*, and *aac(3′)-IIa*, *catB3*, *mphA*, and *arr-3* resistance genes (Table S3). The same isolate contained IncN2 and IncR replicons.

*C. portucalensis* ST493 isolates were positive for IMP-4, obtained from Australia, and contained *qacG2*, *aacA4*, and *catB3*. ST493 isolates were positive for IncA/C, IncFII, IncL, and IncHI2 replicons (Table 1). ST493 also contained *tetD*, *mphE*, *msrE*, *sul1*, *dfrA19*, *dfrA12*, *aph(6′)-1d*, *aadA2*, *bla*_TEM-1_, *bla*_OXA-1_, and *catB3*. The frequency of these resistance determinants was higher in ST493 than in other dominant STs (Table S3).

*C. portucalensis* ST545 isolates were positive for VIM-31 and obtained from Turkey. Four of 5 isolates also harbored *bla*_OXA-48_. All ST545 isolates were positive for IncFIB, IncFII, IncHI1A, IncHI1B, IncL/M and IncN replicons. In comparison to other dominant STs, ST545 had increased frequencies of AMR determinants, including *aadA2*, *dfrA12*, *sul1*, *sul2*, *mphA*, *qnrB17*, and *tetD* (Table S3).

The frequencies of other β-lactamases among *Citrobacter* spp. were as follows: CTX-Ms, 14%; OXAs (non-OXA-48), 35%; TEM-1, 43%; and SHV-12, 14% (Table 1). OXAs were more frequent among ST493 isolates and TEM-1 was more frequent in ST98 and ST493 (Table 1).

### Carbapenemase gene flanking regions.

Due to the limitations of short-read sequencing (7), analyses of the immediate carbapenemase gene flanking regions and plasmids harboring carbapenemase genes were insufficient for some of the isolates. We were able to characterize the immediate carbapenemase gene flanking regions for 16/29 isolates with *bla*_KPC_, 13/13 isolates with *bla*_NDM_, 8/8 isolates with *bla*_OXA-48_, and all the class I integrons containing *bla*_IMP_ (*n* = 20) and *bla*_VIM_ (*n* = 21).

The *bla*_KPC-2_ (*n* = 9) was situated within Tn*4401a* in a single *C. koseri* isolate and within Tn*4401b* in 8 *Citrobacter* species isolates which belonged to different species and STs. The *bla*_KPC-3_ (*n* = 7) was situated within Tn*4401a* (*n* = 3) and Tn*4401b* (*n* = 4) that belonged to different *Citrobacter* species and STs. The *bla*_NDM_ were located on truncated Tn*125* elements and situated downstream of IS*Aba*125 among all the isolates. The *bla*_NDM_ upstream regions showed significant diversities with various IS family insertions (e.g., IS*3*, IS*5*, IS*6*, IS*30* and IS*91*). All the *bla*_OXA-48_ were situated within Tn*1999.2*.

**FIG 3:**
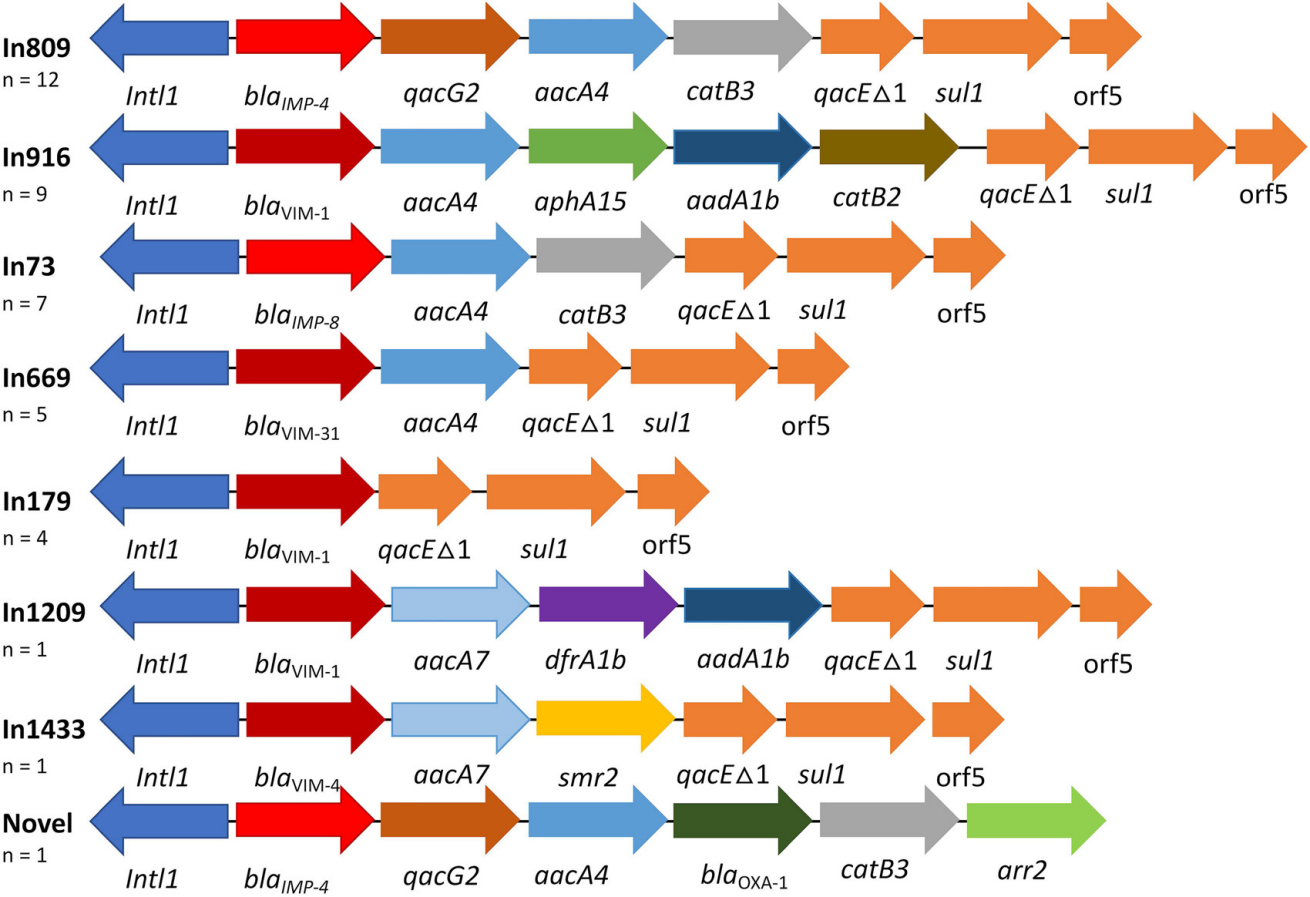
Diagram of integrons detected in carbapenemase-positive *Citrobacter* spp. The array of promoterless gene cassettes is indicated by arrows.

The IMP-4-positive isolates (*n* = 13) were obtained from Australia and consisted of C. freundii ST396 (*n* = 4) and *C. portucalensis* ST493 (*n* = 9). The *bla*_IMP-4_ (12/13) was situated in the class 1 integron In809 that also contained *qacG2*, *aacA4*, *catB3* (Fig. 3). The remaining *bla*_IMP-4_ was situated in a novel class 1 integron that also contained *qacG2*, *aacA4*, *bla*_OXA-1_, *catB3*, and *arr-2* (Fig. 3). Unfortunately, due to the absence of sequence data on the integron 5′ and 3′ ends, a novel integron number was not assigned for this structure.

The IMP-8-positive isolates (*n* = 7) were obtained from Taiwan and consisted of C. freundii STs, namely, ST98 (*n* = 5), ST8 (*n* = 1), and ST528 (*n* = 1). The *bla*_IMP-8_ was situated in the class 1 integron In73 among the different STs. In73 also contained *aacA4* and *catB3* (Fig. 3).

The VIM-1-positive isolates (*n* = 15) consisted of multiple STs (*n* = 10) and were obtained from Italy (*n* = 11), and 1 each from Poland, Portugal, Spain, and the United States. The majority of *bla*_VIM-1_ (*n* = 9) were situated in a class 1 integron In916 and were obtained from Italy (i.e., ST22, ST112, ST523, ST686), Poland (i.e., ST98), and Portugal (i.e., ST95). In916 also contained *aacA4*, *aphA15*, *aadA1b*, and *catB2.* The remaining VIM-1 genes (*n* = 6) were situated in class integrons In179 and In1209 (Fig. 3).

The VIM-31-positive isolates (*n* = 5) were obtained from Turkey and consisted of *C. portucalensis* ST545. The *bla*_VIM-31_ gene was situated in the class 1 integron In669 that also contained *aacA4*. The VIM-4 (*n* = 1) was present in *C. portucalensis* ST63 from Hungary and was situated in In1433 which also contained *aacA7* and *smr2* (Fig. 3).

## DISCUSSION

The KPC-producing *Citrobacter* spp. from this survey were mainly from Colombia (41%) and the United States (31%); VIMs were mainly from Italy (52%) and Turkey (24%), IMPs were mainly from Australia (65%) and Taiwan (35%), NDMs were mainly from the Philippines (46%), and the OXA-48-like carbapenemases were mainly obtained from Turkey (50%). These results are similar to those of previously reported global CPE surveys (8).

VIM and IMP metallo-β-lactamases are rare among CPEs, especially within Klebsiella spp. and E. coli (9). CPE isolates with *bla*_IMP_ are endemic in Japan, Taiwan, and Australia (3, 10) while CPE with *bla*_VIM_ are mainly found in Italy and Greece (4, 11). Among the *Citrobacter* spp. from this study, VIMs and IMPs were the 2nd and 3rd most common carbapenemases. VIMs were found in *Citrobacter* spp. from Turkey and Italy while IMPs were limited to Taiwan and Australia. The Turkish isolates (*n* = 5) were positive for *bla*_VIM-31_ and belonged to a single clone (*C. portucalensis* ST545) that contained the class I integron In669. Italian isolates (*n* = 11) were positive for *bla*_VIM-1_, belonged to multiple C. freundii STs, and contained In916. In916 was also found in *Citrobacter* spp. from Poland (ST98) and Portugal (ST95). This indicated that identical class I integrons with *bla*_VIM-1_ are circulating between different STs in Italy, Poland, and Portugal. A similar scenario was previously reported with Enterobacter spp. harboring *bla*_VIM-1_ within In916 from Spain, Greece, and Italy (12). The IMP-8 isolates from Taiwan belonged to ST8, ST98, and ST528 that harbored the identical class I integron In73. The Australian IMP-4-positive *Citrobacter* species isolates belonged to 2 clones, namely, ST396 and ST493, which contained the identical class I integron In809. This in-depth characterization of AMR isolates showed how genomic surveillance using whole-genome sequencing offered an unprecedented level of detail that clarified the underlying differences among CPEs from various countries.

The geographical distribution and carbapenemase types of the *Citrobacter* spp. from this study were different from those of carbapenemase-producing E. coli obtained from the same surveillance programs over identical periods (2). The most common carbapenemases from the E. coli survey were OXA-181 and NDM-5, while VIMs and IMPs were rare. The *Citrobacter* spp. results from this survey showed some similarities with carbapenemase-producing K. pneumoniae (13) and Enterobacter cloacae complex (12) obtained from the same surveillance programs. The K. pneumoniae with KPC-2 was obtained mainly from Colombia and the isolate with KPC-3 was mainly from the United States. The E. cloacae complex isolates consisted mainly of VIM-1 isolates obtained from Italy and Greece. *Citrobacter* spp., K. pneumoniae (1), and Enterobacter spp. (14) are typically hospital pathogens, while E. coli is typically a community pathogen (15). This fact could be partly responsible for the different carbapenemase types and geographical distributions among these species.

C. freundii (60%) and *C*. *portucalensis* (23%) were the principal species among *Citrobacter* spp. with carbapenemases from this survey. *C*. *portucalensis* was first reported in 2017 from Portuguese aquatic samples (16) and later from Nigerian leafy vegetables (17). This bacterium is closely related to and often misidentified as C. freundii, especially when using phenotypic identification systems such as Vitek 2.0 and MALDI-TOF MS (18). *C*. *portucalensis* with *bla*_CTX-M-15_ (19) and with *bla*_NDM-1_ (18) were previously reported from Brazil (two environmental isolates) and China (one clinical isolate), respectively. In our study, MALDI-TOF MS identified *C*. *portucalensis* as C. freundii. *C. portucalensis* was mainly found in Australia and Turkey, but also in Egypt, Hungary, Italy, Jordan, and the Philippines. The C. freundii population was linked with various STs obtained from Colombia, the United States, and Italy. The population structure of *C. portucalensis* consisted mainly of two clones, namely, ST493 with IMP-4 (Australia) and ST545 with VIM-31 (Turkey). Our results indicate that the geographical and underlying molecular epidemiology is different between carbapenemase-producing C. freundii and *C. portucalensis* isolates. Therefore, future surveys should use methodologies that can distinguish between C. freundii and *C. portucalensis* isolates. This will emphasize the role of *C. portucalensis* in the global dissemination of carbapenemases among *Citrobacter* spp. We recommend that identification systems in clinical laboratories be updated to include the routine identification of *C. portucalensis.* This will enable the clinical microbiology community to determine the overall prevalence, clinical significance, and geographical distribution of this newly described *Citrobacter* species.

High-risk AMR clones such as K. pneumoniae ST258 (3) and E. coli ST410 (20) contribute significantly to the global spread of CPE. We observed a high diversity of carbapenemase-producing *Citrobacter* spp. clones that was represented by 37 STs, including 4 dominant clones representing >40% of the total *Citrobacter* spp. population. C. freundii ST98 and ST22 were global, whereas C. *portucalensis* ST493 and ST545 were limited to Australia and Turkey, respectively. The dominant STs were linked to different carbapenemase genes. The global carbapenemase *Citrobacter* spp. population is dominated by diverse STs with different characteristics and varied geographical distributions. ST22 and ST98 isolates with different carbapenemases (VIMs, KPC-2, NDM-1, OXA-48) were previously reported from Spain (21, 22), Tunisia (23) and Germany (24).

We provide pertinent information about the global distribution of carbapenemases and population structure among a large collection of carbapenemase-producing *Citrobacter* spp. This study has some limitations. We used short-read sequencing to characterize our collection, which limited our ability to fully reconstruct flanking regions and plasmids harboring carbapenemases. Additionally, many countries contributed few isolates, and statistical approaches to deal with clustered data failed to estimate country-adjusted rates of carbapenemases among *Citrobacter* spp. Countries contributing few isolates may not be fully representative of the population of carbapenemases in *Citrobacter* spp. from that region. The global distribution and prevalence of carbapenemases will be influenced by the clonal dissemination of isolates during nosocomial outbreaks that might have occurred during the surveillance period.

In summary, carbapenemase-producing *Citrobacter* spp. are a highly diverse group of bacteria that contain 4 dominant global clones. Carbapenemase-producing *Citrobacter* spp. are disseminated either clonally and polyclonally depending on the species, ST, and geographical location. We also demonstrated that the newly described *C. portucalensis* has a global distribution and is likely involved in some of the clonal outbreaks of carbapenemase-producing *Citrobacter* spp. Our findings greatly contribute to global and local surveillance activities, particularly those in lower- and middle-income countries. Our findings underline the increasing importance of *Citrobacter* spp. in the global dissemination of carbapenemases.

## MATERIALS AND METHODS

### Bacterial isolates.

We obtained isolates from two global surveillance programs (2015 to 2017), namely, the Merck Study for Monitoring Antimicrobial Resistance Trends (SMART) and the International Network for Optimal Resistance Monitoring (INFORM) programs. The SMART program includes Gram-negative isolates from intra-abdominal, lower respiratory tract, and urinary tract infections obtained from 55 countries, whereas the INFORM program collects isolates from blood, intra-abdominal, lower respiratory tract, skin, soft tissue, and urinary tract infections obtained from 42 countries. Participating countries are listed in the Appendix (Table S1). Medical centers contributed isolates regardless of their antimicrobial resistance profiles.

The two programs collect around 100 consecutive, nonrepetitive, and clinically relevant (based on local definitions) Gram-negative bacteria per year at each participating center. The isolates underwent phenotypic identification using MALDI-TOF MS (Vitek AMS; bioMérieux Vitek Systems Inc., Hazelwood, MO) and custom microdilution panel susceptibility testing using Clinical Laboratory and Standards Institute guidelines, as described previously (9, 25, 26). Overall, 87,182 Enterobacterales isolates were obtained from 2015 to 2017, of which 2,256 (2.6%) were identified as *Citrobacter* spp., and 112 (5%) tested nonsusceptible to meropenem (MIC ≥ 2 μg/mL). Meropenem-nonsusceptible *Citrobacter* spp. were screened for presence of carbapenemase genes (i.e., KPCs, NDMs, OXA-48-like, IMPs, VIMs, and GES), as described previously (9, 26). *Citrobacter* species isolates that were positive for carbapenemases (*n* = 86) were included in this study.

“Dominant” sequence types were defined as those representing >5% of the total population of *Citrobacter* spp. with carbapenemases (27).

### Genomic analysis.

Carbapenemase-producing *Citrobacter* species isolates were sequenced on an Illumina NovaSeq SP with a 300-cycle run, 2 × 150 paired-end reads, and a minimum of 100× coverage. SPAdes 3.15 (28) *de novo* assembly generated draft genomes. TYGS (the Type [Strain] Genome Server) (29) and an average nucleotide identity (ANI) calculator (30) were used for species identification. The ANI cutoff score for genomes belonging to the same species was ≥95%. ResFinder 4.1 (31), PlasmidFinder 2.1 (32), and INTEGRALL (33), were accessed to identify antimicrobial resistance genes, plasmid replicons, and integron types in draft genomes, respectively. Multilocus sequence typing was done *in silico* at PubMLST (34). A whole-genome SNP-based phylogeny was built using kSNP 3.0 (35). The following reference genomes were included in our analysis: C. freundii ATCC 8090, *C. portucalensis* NCTC11104, *C. braakii* FDAARGOS_1421, *C. koseri* ATCC BAA-895, *C. farmeri* FDAARGOS_1423, and *C. amalonaticus* FDAARGOS_122. *C. werkmanii* UMH18 was selected as the out-species for this analysis.

### Statistical analysis.

We used frequency tables to summarize presence of antimicrobial resistance genes, integron types, and plasmid replicon types for each dominant ST. Initially, we attempted to fit exact logistic regression models for clustered data considering isolates clustered within countries, but the models failed to converge. We then used Fisher’s exact tests to perform pairwise comparisons of each outcome between dominant STs. We adjusted *P* values for multiple comparisons for each individual outcome using the false discovery rate (36). We considered statistical significance at the 5% level. We used R version 4.1.2 for all analyses.

### Ethics statement.

Ethics approval for this study was obtained through the University of Calgary Conjoint Health Research Ethics Board (REB17-1010).

### Data availability.

The sequencing data were deposited in the NCBI database (BioProject ID PRJNA882828).
