# Supplementary material for: Molecular Epidemiology of Global Carbapenemase-Producing Citrobacter spp. (2015–2017)
Source: Microbiol Spectr. 2023 Feb 27;11(2):e04144-22. doi: 10.1128/spectrum.04144-22 (PMC10101073; doi:10.1128/spectrum.04144-22)
Supplement: Supplemental file 1 — Tables S1 to S3. Download spectrum.04144-22-s0001.pdf, PDF file, 0.1 MB [file spectrum.04144-22-s0001.pdf]

**SUPPLEMENTAL MATERIAL**

**TABLE S1** List of countries included in the SMART and INFORM surveillance programs

| Program[1] | Countries |
|---|---|
| SMART | Argentina, Australia, Brazil, Canada, Chile, China, Colombia, Croatia, Czech Republic, Dominican Republic, Ecuador, Egypt, Estonia, France, Georgia, Germany, Greece, Guatemala, Hong Kong, Hungary, India, Israel, Italy, Japan, Jordan, Kenya, Latvia, Lebanon, Lithuania, Malaysia, Mexico, Morocco, New Zealand, Panama, Philippines, Portugal, Puerto Rico, Romania, Saudi Arabia, Serbia, Singapore, Slovenia, South Africa, South Korea, Spain, Switzerland, Taiwan, Thailand, Tunisia, Turkey, UAE, United Kingdom, United States, Uruguay, Venezuela, Vietnam |
| INFORM | Argentina, Australia, Austria, Belgium, Brazil, Bulgaria, Chile, China, Colombia, Czech Republic, Denmark, Egypt, France, Germany, Greece, Hungary, Israel, Italy, Japan, Kenya, Kuwait, Lebanon, Macedonia, Mexico, Nigeria, Philippines, Poland, Portugal, Romania, Russia, Slovakia, South Africa, South Korea, Spain, Syria, Taiwan, Thailand, Turkey, United Kingdom, United States, Uruguay, Venezuela |

[1]Each program includes around 100 consecutive, non-repetitive Gram-negative bacteria per year at each participating centre

**TABLE S2** Carbapenemase-producing *Citrobacter* spp. (n=86): species, geographic location, and Sequence Types.

| Carbapenemase (n) | Species (n) | Geographic location (n) | Sequence Types (n) |
|---|---|---|---|
| KPCs (28) | | | |
| KPC-2 (22) | *C. freundii* (14), *C. koseri* (6), *C. portucalensis* (2) | Colombia (11), United States (5), Argentina (3), Italy (2), Brazil (1) | ST22 (4), ST11 (3), ST900 (3), ST63 (2), ST98 (2), ST535 (2), ST544 (2), ST415 (1), ST899 (1), ST902 (1), ST903 (1) |
| KPC-3 (6) | *C. freundii* (5), *C. koseri* (1) | United States (4), Austria (1), Colombia (1) | ST22 (2), ST98 (1), ST111 (1), ST527 (1), ST901 (1) |
| IMPs (20) | | | |
| IMP-4 (13) | *C. portucalensis* (9), *C. freundii* (4) | Australia (13) | ST493 (9), ST396 (4) |
| IMP-8 (7) | *C. freundii* (7) | Taiwan (7) | ST98 (5), ST8 (1), ST528 (1) |
| VIMs (16) | | | |
| VIM-1 (14) | *C. freundii* (12), *C. farmeri* (2) | Italy (10), Poland (1), Portugal (1), Spain (1), United States (1) | ST112 (4), ST22 (3), ST98 (2), ST686 (2), ST91 (1), ST95 (1), ST415 (1), ST545 (1) |
| VIM-4 (1) | *C. portucalensis* (1) | Hungary (1) | ST63 (1) |
| VIM-31 (1) | *C. portucalensis* (1) | Turkey (1) | ST545 (1) |
| NDMs (13) | | | |
| NDM-1 (9) | *C. freundii* (5), *C. portucalensis* (2), *C. farmeri* (1), *C. koseri* (1) | Philippines (3), Thailand (3), Egypt (1), Jordan (1), South Africa (1) | ST116 (2), ST18 (1), ST21 (1), ST22 (1), ST85 (1), ST549 (1), ST686 (1), ST690 (1) |
| NDM-5 (1) | *C. koseri* (1) | Australia (1) | ST854 (1) |
| NDM-7 (3) | *C. freundii* (2), *C. portucalensis* (1) | Philippines (3) | ST98 (1), ST214 (1), ST539 (1) |

| | | | |
|---|---|---|---|
| OXA-48 (4) | *C. amalonaticus* (1), *C. braakii* (1), *C. freundii* (1), *C. koseri* (1) | Belgium (1), Italy (1), Serbia (1), Spain (1) | ST548 (1), ST550 (1), ST710 (1), ST904 (1) |
| Two carbapenemases | | | |
| KPC-3 + VIM-1 (1) | *C. freundii* (1) | Italy (1) | ST523 (1) |
| OXA-48 + VIM-31 (4) | *C. portucalensis* (4) | Turkey (4) | ST545 (4) |

**TABLE S3** Resistance determinants detected in dominant Sequence Types among carbapenemase-positive *Citrobacter* spp.

| **Resistance determinants** | **ST98 (n = 11)** | **ST22 (n = 10)** | **ST493 (n = 9)** | **ST545 (n = 5)** | **Other STs[1] (n = 51)** | **All STs (n = 86)** |
|---|---|---|---|---|---|---|
| Aminoglycoside modifying enzymes: | | | | | | |
| *aac(3')-IIa* | 0 (0%) | 2 (20%) | 0 (0%) | 0 (0%) | 5 (9.8%) | 7 (8.1%) |
| *aac(6')-Ib-cr* | 7 (63.6%)[a] | 8 (80%)[a] | 1 (11.1%)[b] | 0 (0%)[b] | 27 (52.9%) | 43 (50%) |
| *aac(6')-Ib* | 1 (9.1%) | 0 (0%) | 0 (0%) | 0 (0%) | 2 (3.9%) | 3 (3.5%) |
| *aadA1* | 5 (45.5%)[a,c] | 6 (60%)[a] | 0 (0%)[b,c] | 0 (0%)[c] | 17 (33.3%) | 28 (32.6%) |
| *aadA2* | 0 (0%)[a] | 1 (10%)[a] | 9 (100%)[b] | 5 (100%)[b] | 3 (5.9%) | 18 (20.9%) |
| *aadA5* | 0 (0%) | 0 (0%) | 0 (0%) | 0 (0%) | 1 (2%) | 1 (1.2%) |
| *aph(3')-Ib* | 5 (45.5%)[a] | 0 (0%)[b] | 0 (0%)[b] | 0 (0%) | 3 (5.9%) | 8 (9.3%) |
| *aph(3')-XV* | 1 (9.1%) | 3 (30%) | 0 (0%) | 0 (0%) | 6 (11.8%) | 10 (11.6%) |
| *aph(6')-1d* | 4 (36.4%)[a] | 0 (0%)[a] | 9 (100%)[b] | 0 (0%)[a] | 8 (15.7%) | 21 (24.4%) |
| *armA* | 0 (0%) | 0 (0%) | 0 (0%) | 0 (0%) | 1 (2%) | 1 (1.2%) |
| *rmtC* | 0 (0%) | 0 (0%) | 0 (0%) | 0 (0%) | 1 (2%) | 1 (1.2%) |
| *rmtD2* | 0 (0%) | 0 (0%) | 0 (0%) | 0 (0%) | 1 (2%) | 1 (1.2%) |
| Other AMR determinants: | | | | | | |
| *arr-2* | 0 (0%) | 0 (0%) | 0 (0%) | 0 (0%) | 4 (7.8%) | 4 (4.7%) |
| *arr-3* | 1 (9.1%) | 5 (50%) | 3 (33.3%) | 0 (0%) | 6 (11.8%) | 15 (17.4%) |
| *catB2* | 1 (9.1%) | 3 (30%) | 0 (0%) | 0 (0%) | 6 (11.8%) | 10 (11.6%) |
| *catB3* | 7 (63.6%)[a,b] | 2 (20%)[b,c] | 8 (88.9%)[a] | 0 (0%)[c] | 11 (21.6%) | 28 (32.6%) |
| *dfrA1* | 0 (0%) | 1 (10%) | 0 (0%) | 0 (0%) | 3 (5.9%) | 4 (4.7%) |
| *dfrA12* | 0 (0%)[a] | 1 (10%)[a] | 9 (100%)[b] | 5 (100%)[b] | 3 (5.9%) | 18 (20.9%) |
| *dfrA14* | 4 (36.4%) | 3 (30%) | 0 (0%) | 0 (0%) | 10 (19.6%) | 17 (19.8%) |
| *dfrA16* | 0 (0%) | 0 (0%) | 0 (0%) | 0 (0%) | 1 (2%) | 1 (1.2%) |
| *dfrA17* | 0 (0%) | 0 (0%) | 0 (0%) | 0 (0%) | 1 (2%) | 1 (1.2%) |
| *dfrA19* | 1 (9.1%)[a] | 0 (0%)[a] | 9 (100%)[b] | 0 (0%)[a] | 1 (2%) | 11 (12.8%) |
| *dfrA27* | 0 (0%) | 3 (30%) | 0 (0%) | 0 (0%) | 3 (5.9%) | 6 (7%) |
| *dfrA29* | 0 (0%) | 0 (0%) | 0 (0%) | 0 (0%) | 2 (3.9%) | 2 (2.3%) |

| | | | | | | |
|---|---|---|---|---|---|---|
| *dfrB1* | 1 (9.1%) | 0 (0%) | 0 (0%) | 0 (0%) | 0 (0%) | 1 (1.2%) |
| *sul1* | 8 (72.7%) | 7 (70%) | 9 (100%) | 5 (100%) | 31 (60.8%) | 60 (69.8%) |
| *sul2* | 9 (81.8%)[a] | 5 (50%)[a] | 0 (0%)[b] | 5 (100%)[a] | 12 (23.5%) | 31 (36%) |
| *msrE* | 2 (18.2%)[a] | 0 (0%)[a] | 9 (100%)[b] | 0 (0%)[a] | 2 (3.9%) | 13 (15.1%) |
| *mphA* | 6 (54.5%) | 9 (90%)[a] | 2 (22.2%)[b] | 5 (100%)[a] | 18 (35.3%) | 40 (46.5%) |
| *mphE* | 1 (9.1%)[a] | 0 (0%)[a] | 9 (100%)[b] | 0 (0%)[a] | 2 (3.9%) | 12 (14%) |
| *qnrA1* | 2 (18.2%) | 0 (0%) | 0 (0%) | 0 (0%) | 0 (0%) | 2 (2.3%) |
| *qnrB1* | 0 (0%) | 0 (0%) | 0 (0%) | 0 (0%) | 1 (2%) | 1 (1.2%) |
| *qnrB2* | 1 (9.1%)[a] | 0 (0%)[a] | 5 (55.6%)[b] | 0 (0%) | 4 (7.8%) | 10 (11.6%) |
| *qnrB4* | 0 (0%) | 1 (10%) | 0 (0%) | 0 (0%) | 2 (3.9%) | 3 (3.5%) |
| *qnrB6* | 0 (0%) | 0 (0%) | 0 (0%) | 0 (0%) | 2 (3.9%) | 2 (2.3%) |
| *qnrB17* | 0 (0%)[a] | 0 (0%)[a] | 0 (0%)[a] | 5 (100%)[b] | 0 (0%) | 5 (5.8%) |
| *qnrB35* | 0 (0%) | 0 (0%) | 0 (0%) | 0 (0%) | 3 (5.9%) | 3 (3.5%) |
| *qnrB38* | 0 (0%) | 0 (0%) | 0 (0%) | 0 (0%) | 2 (3.9%) | 2 (2.3%) |
| *qnrS1* | 2 (18.2%) | 0 (0%) | 0 (0%) | 0 (0%) | 4 (7.8%) | 6 (7%) |
| *qnrS2* | 1 (9.1%) | 0 (0%) | 0 (0%) | 0 (0%) | 0 (0%) | 1 (1.2%) |
| *tetA* | 0 (0%) | 2 (20%) | 0 (0%) | 0 (0%) | 4 (7.8%) | 6 (7%) |
| *tetB* | 0 (0%) | 0 (0%) | 0 (0%) | 0 (0%) | 2 (3.9%) | 2 (2.3%) |
| *tetD* | 0 (0%)[a] | 6 (60%)[b] | 9 (100%)[b] | 5 (100%)[b] | 0 (0%) | 20 (23.3%) |

[a-c]Rates followed by different letters indicate significant differences between dominant STs at the 5% level (adjusted for multiple comparisons for each outcome).

[1]Other STs (n) include the following: ST112 (4), ST396 (4), ST11 (3), ST63 (3), ST686 (3), ST900 (3), ST116 (2), ST415 (2), ST535 (2), ST544 (2), ST8 (1), ST18 (1), ST21 (1), ST85 (1), ST91 (1), ST95 (1), ST111 (1), ST214 (1), ST523 (1), ST527 (1), ST528 (1), ST539 (1), ST548 (1), ST549 (1), ST550 (1), ST690 (1), ST710 (1), ST854 (1), ST899 (1), ST901 (1), ST902 (1), ST903 (1), ST904 (1).
